# *Arabidopsis* DNA polymerase lambda mutant is mildly sensitive to DNA double strand breaks but defective in integration of a transgene

**DOI:** 10.3389/fpls.2015.00357

**Published:** 2015-05-27

**Authors:** Tomoyuki Furukawa, Karel J. Angelis, Anne B. Britt

**Affiliations:** ^1^Department of Plant Biology, University of California at DavisDavis, CA, USA; ^2^DNA Repair Lab, Institute of Experimental Botany of the Academy of Sciences of the Czech RepublicPraha, Czech Republic

**Keywords:** DNA polymerase, DNA repair, Non homologous end joining, DNA damage response, double strand breaks

## Abstract

The DNA double-strand break (DSB) is a critical type of damage, and can be induced by both endogenous sources (e.g., errors of oxidative metabolism, transposable elements, programmed meiotic breaks, or perturbation of the DNA replication fork) and exogenous sources (e.g., ionizing radiation or radiomimetic chemicals). Although higher plants, like mammals, are thought to preferentially repair DSBs via nonhomologous end joining (NHEJ), much remains unclear about plant DSB repair pathways. Our reverse genetic approach suggests that DNA polymerase λ is involved in DSB repair in *Arabidopsis*. The *Arabidopsis* T-DNA insertion mutant (*atpolλ-1*) displayed sensitivity to both gamma-irradiation and treatment with radiomimetic reagents, but not to other DNA damaging treatments. The *atpolλ-1* mutant showed a moderate sensitivity to DSBs, while *Arabidopsis* Ku70 and DNA ligase 4 mutants (*atku70-3* and *atlig4-2*), both of which play critical roles in NHEJ, exhibited a hypersensitivity to these treatments. The *atpolλ-1*/*atlig4-2* double mutant exhibited a higher sensitivity to DSBs than each single mutant, but the *atku70*/*atpolλ-1* showed similar sensitivity to the *atku70-3* mutant. We showed that transcription of the *DNA ligase 1, DNA ligase 6*, and *Wee1* genes was quickly induced by BLM in several NHEJ deficient mutants in contrast to wild-type. Finally, the T-DNA transformation efficiency dropped in NHEJ deficient mutants and the lowest transformation efficiency was scored in the *atpolλ-1*/*atlig4-2* double mutant. These results imply that AtPolλ is involved in both DSB repair and DNA damage response pathway.

## Introduction

The 3R mechanisms (DNA replication, repair, and recombination) are key machineries for all living organisms. DNA-dependent DNA polymerases play critical roles in 3R mechanisms. To date, at least 13 types of DNA polymerases (Pol α, β, γ, δ, ε, ζ, η, θ, ι, κ, λ, μ, and ν) and two polymerase homologs, terminal deoxyribonucleotidyl transferases (TdT), and REV1, have been found in the human genome. Based on amino acid sequence homology, DNA polymerases are classified into four different polymerase families (A, B, X, and Y). DNA polymerase λ belongs to the Pol X-family along with three other non-replicative mammalian DNA polymerases (Pol β, Pol μ, and TdT). The structure of the Pol λ protein consists of three functional domains: a BRCT (BRCA1 C-terminus) domain at the N-terminus, a DNA binding domain in the central region, and a DNA polymerization domain at the C-terminus, respectively. Biochemical studies have revealed that the human Pol λ protein has three enzymatic activities: DNA polymerase activity, TdT activity, and 5′-Deoxyribose-5-phosphate (dRP lyase) activity. Although the human Pol λ protein is able to incorporate multiple nucleotides during the *in vitro* reaction, its processivity is low compared to replicative-type DNA polymerases (Pol α, δ, ε). These enzymatic activities suggest that Pol λ participates in two DNA repair pathways; base excision repair and NHEJ (Braithwaite et al., 2005a,b, 2010; Garcia-Diaz et al., 2005; Nick McElhinny et al., 2005). Both DNA polymerase and dRP lyase activities are required for short-patch base excision repair (spBER). Physical interaction of Pol λ with the XRCC4/Lig4 complex implies that Pol λ also participates in alignment-based gap filling during NHEJ (Fan and Wu, 2004; Lee et al., 2004; Capp et al., 2006).

In contrast to a long history of study of mammalian and yeast DNA polymerases, much remains unclear regarding the plant DNA polymerases. A recent advance of plant genome projects has revealed that plant genomes encode homologs for 10 DNA polymerases (Pol α, δ, ε, ζ, η, θ, κ, λ, σ, and ν) (Yokoi et al., 1997; Uchiyama et al., 2002, 2004; Sakamoto et al., 2003; Garcia-Ortiz et al., 2004; Takahashi et al., 2005; Inagaki et al., 2006; Anderson et al., 2008) and two plastid-specific Pol I-like DNA polymerases (Kimura et al., 2002; Mori et al., 2005; Ono et al., 2007). Despite the fact that Pol λ is widely conserved in the genome regardless of higher (*Oryza sativa* as monocots and *Arabidopsis* as dicots) and lower (*Chlamydomonas reinhardtii*, Uchiyama et al., 2009) plants, no other Pol X-family homolog genes have been identified in plant genomes. These observations strongly indicate that plants have only Pol λ among Pol X-family members.

The function of plant Pol λ has been studied using rice and *Arabidopsis* as model plants (Garcia-Diaz et al., 2000; Uchiyama et al., 2004; Amoroso et al., 2011; Roy et al., 2011). Like human Pol λ, rice Pol λ protein (OsPol λ) possesses DNA polymerase activity, weak TdT activity, and dRP lyase activity and this polymerase activity is activated by rice PCNA (proliferating cell nuclear antigen) protein. Expression analysis of *OsPol λ* transcripts suggests that it functions in DNA replication and/or repair in both meristematic and meiotic tissues (Uchiyama et al., 2004). The physical partner of plant Pol λ protein has been identified by yeast two–hybrid or pull-down assay. OsPol λ is able to bind with rice exonuclease-1, but not with rice XRCC1 (Furukawa et al., 2008; Uchiyama et al., 2008). Physical interaction of AtPol λ with AtPCNA2 stimulates its fidelity and efficiency in translesion synthesis (Amoroso et al., 2011). The role of *Arabidopsis Pol λ* (*AtPol λ*) in DNA repair has been recently reported. UV-B radiation induces the expression of *AtPol λ*, and three *AtPol λ* mutants (*atpolλ-1, atpolλ-2*, and *atpolλ-3*) exhibit sensitivity to UV-B and MMC (Roy et al., 2011, 2013). The *AtPol λ* mutants show increased sensitivity when exposed to high salinity and MMC treatment. AtPol λ is able to interact with AtLig4 and AtXRCC4 through its BRCT domain and *atpolλ-2*/*atxrcc4* and *atpolλ-2*/*atlig4* double mutants show delayed repair of salinity-induced DSBs (Roy et al., 2013). These findings suggest that plant Pol λ plays a role in various DNA repair pathways. Recent studies have indicated a role for Pol λ in the repair of transposable element excision sites, suggesting involvement in the repair of DSBs (Huefner et al., 2011).

We report here that *Arabidopsis* DNA polymerase λ (*AtPol λ*) is employed in DSB repair in response to clastogenic agents and is involved in T-DNA integration. In addition, our results imply that AtPol λ, in concert with AtLig1 and AtLig6, may participate in the Lig4-independent alternative NHEJ pathway.

## Materials and methods

### Isolation of mutants

We used the *Arabidopsis thaliana* parental strain ecotype Col (Columbia) in this study. The *atpolλ-1* (SALK_75391C) and *atku70-3* (SALK_123114C) mutants were identified using the Salk SIGnAL Web site (http://signal.salk.edu/), and their seeds were obtained from the ABRC. The *atlig4-2* line has been previously described (Friesner and Britt, 2003). The *atpolλ-1* and *atku70* homozygous mutants were identified by genotyping PCR with gene-specific primer sets. To analyze the *atpolλ-1* transcript, PCR with three different primer sets (polλ-1AF + polλ-1AR, polλ-1BF + polλ-1BR, and polλ-1CF + polλ-1CR) were performed with cDNA synthesized from *atpolλ-1* total RNA. The upstream region of the *atpolλ-1* transcript containing a RB border region was amplified by PCR with the polλ-1BF + RBc1 primer set and the amplified PCR product was sequenced using the RBb1 primer. The downstream region containing a LB border was amplified with the LBb1 + polλ-1CR primer set and the amplified product was sequenced using the LBb1 primer. The *atpolλ-1* line was crossed with either *atku70-3* or *atlig4-2* line to make double mutants. Homozygous F2 offsprings were screened by genotyping PCR with gene specific primer sets; polλ −1BF, polλ-1BR, ku70F, ku70R and T-DNA specific primer LBb1 for *atku70-3*/*atpolλ-1* double mutants and polλ-1BF, polλ-1BR, lig4-2B, lig4-2C, and LBb1 for *atpolλ-1*/*atlig4-2* double mutants. Primers sequences were shown in Supplemental Table S1.

### Growth of *Arabidopsis*

Seeds in microcentifuge tubes were surface-sterilized in an air-tight container filled with chlorine gas for 2 h. Chlorine gas was produced by mixing 30 ml of bleach and 5 ml of hydrochloric acid. Following degassing of chlorine gas in a fume hood, sterilized seeds were imbibed in water for 2 days at 4°C. The sterilized seeds were then sown on solid 1 × Murashige and Skoog (MS, Sigma-Aldrich, St. Louis, MO, USA) with pH adjusted to 5.8 using 1N KOH containing 0.8% phytoagar (PlantMedia, Dublin, OH, USA) plates or on soil, and grown in a climate chamber under cool-white lamps at an intensity of 100–150 μmol m^−2^ s^−1^ with a cycle of 16 h day/8 h night at 20°C.

### DNA damaging treatments

For sensitivity tests to DNA damaging reagents such as methyl methanesulfonate (MMS, Fisher Scientific, Pittsburgh, PA, USA), mitomycin C (MMC, Fisher), methyl viologen (MV, Fisher), and bleomycin (BLM, Bleocin inj., Euro Nippon Kayaku GmbH, Germany), chlorine gas-sterilized seeds were sown on solid MS-agar plates supplemented with each chemical. Seeds were grown for 7 days in a growth chamber under the normal growth condition as shown above. For the root-swollen assay, the 3 day-old seedlings were transferred to MS-agar plates containing 0.1 or 0.25 μg mL^−1^ bleomycin and grown in a growth chamber under the normal condition. Ultraviolet B (UV-B) irradiation was performed according to Jiang et al. (1997). Sterilized seeds were planted on solid 1 x MS medium and grown with the plate oriented vertically for 3 days as described above. Seedlings were irradiated with UV-B in the absence of visible light using a UV-transilluminator (Fisher) filtered with 0.005 ml cellulose acetate membrane, with a flux rate of 5.5 mW cm^−2^. The UV-B irradiated plates were rotated by 90°, then were cultivated for two more days under orange light to prevent photoreactivation. To investigate sensitivity of mutants to γ-irradiation, chlorine gas-sterilized seeds in water were γ-irradiated at 0, 40, 60, 80, and 100 Gy (6.43 Gy min^−1^) from a ^137^Cs source (Institute of Toxicology and Environmental Health, University of California, Davis). Gamma-irradiated seeds were sown on soil or MS-agar plates and grown in a growth chamber as described above.

### Gamma-irradiation and detection of cell death in irradiated *Arabidopsis* plants

Observation of cell death in gamma-irradiated *Arabidopsis* roots were performed as described in Furukawa et al. (2010). Briefly, 5 day-old seedlings on MS-agar plates were γ-irradiated to a final dose of 20 Gy (6.43 Gy min^−1^). Dead cells were visualized by staining of roots with 5 μg mL^−1^ propidium iodide (PI, Sigma-Aldrich) and were observed using a Leica TSC SP2 confocal microscope.

### Quantitative RT-PCR

Prior to treatment, 5 day-old seedlings were gently transferred from agar to MS liquid medium (±30 μg mL^−1^ bleomycin in 5 cm petri plates. The bleomycin treatment time for the expression analysis of DDR genes was 1.5 h, while that of DNA repair genes was 1 h. Treated seedlings were thoroughly rinsed in sterilized water and placed on solid MS plates. Seedlings were collected at each time point after wash. Mock seedlings were treated with liquid MS medium.

Total RNA was extracted from 100 mg of untreated, treated and recovered *Arabidopsis* seedlings collected using the RNeasy kit (Qiagen, Hilden, Germany) according to the manufacturer's protocol. The cDNAs from 1 μg of total RNA were synthesized using iScript cDNA Synthesis kit (Bio-Rad, Hercules, USA) with the help of oligo (dT) blend and random hexamer primers in a 20 μl reaction according to supplied protocol. One microliter of heat denaturated cDNA reaction mixture was used for quantitative RT-PCR assay in 20 μl reaction volume using iTaq SYBR Green Supermix with ROX master mix (Bio-Rad) with the following primers at final concentration of 500 nM.

PCR amplification was carried with LightCycler 480 (Roche, Basel, Switzerland) or MX 3005P cycler (Stratagene, La Jolla, USA). For the reaction with LightCycler 480, an initial denaturation step was for 95°C, 5 min and subsequent 45 cycles of PCR amplification proceeded as follows: denaturation 20 s at 95°C; annealing 20 s at 59°C; extension 30 s at 72°C. For the reaction with MX3005P cycler, an initial denaturation step was for 95°C, 3 min and subsequent 40 cycles of PCR amplification proceeded as follows: denaturation 15 s at 95°C; annealing 40 s at 55°C; extension 40 s at 72°C. After amplification, all fluorescence data were analyzed by the supplied software and normalized against *AtUBQ10* and *AtActin2*, or *AtROC3* reference gene transcripts. Sequences of primers used for qRT-PCR were listed in Supplemental Table S1.

### Plant transformation and observation of fluorescent seeds

The pFLUAR101 fluorescent binary vector (Stuitje et al., 2003) was used to calculate transformation efficiency and transformation of *Arabidopsis* plants was performed by the *Agrobacterium*-mediated floral dip method. All siliques, flowers, and buds whose stage was later than stage 12 were trimmed from plants 1 day before transformation. The pFLAR101 vector was transformed into *Agrobacterium tumefaciens* strain GV3101 by electroporation. *Agrobacterium* transformant cells were cultured in liquid LB medium supplemented with kanamycin overnight at 30°C to reach stationary phase. Following centrifugation, the *Agrobacterium* cells were diluted to an OD600 of 1.8 with 5% sucrose solution. Silwet L-77 was added to a final concentration of 0.02% immediately before dipping. Second trimming was carried out 10 days after dipping to hold 12 siliques seeded from young buds that escaped from first trimming per brunch, and thereafter trimmed bolts were bagged in a glassine paper. Seeds were harvested 3 weeks after second trimming by collecting them in each glassine paper. Transformant seeds expressing fluorescence were screened by the Zeiss SteREO Discovery V12 microscope with a fluorescent filter for DsRED. The number of T_0_ plants, harvested T_1_ seeds, T_1_ seeds expressing DsRED fluorescence, and each plant's transformation efficiency over three trials are described in Supplementary Table S3.

### Statistical analysis

Experimental results were examined using either *t*-test or One-Way ANOVA (analysis of variance) depending the number of samples. The *post-hoc* test (Tukey's HSD) was also used to find which means were significantly different. A *P*-value less than 0.05 (^*^P < 0.05) and 0.01 (^**^P < 0.01) was considered significant.

## Results

### Identification of the *Arabidopsis pol* λ mutant

**Figure 1:**
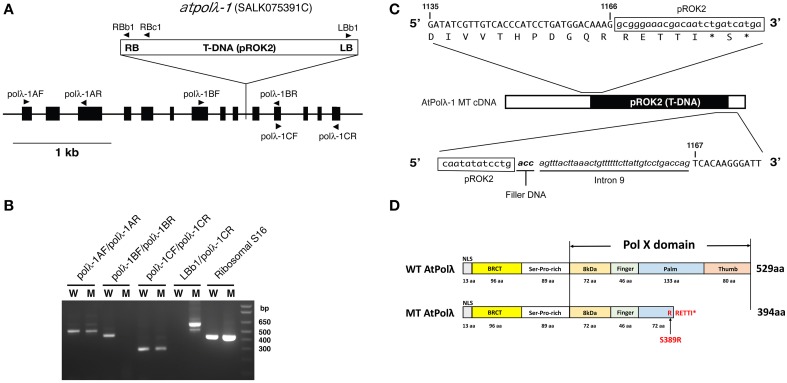
**Structure of DNA polymerase λ gene in the** ***atpolλ-1*** **mutant**. **(A)** Schematic structure of the *AtPolλ* gene and its T-DNA insertion. Arrowheads represent gene-specific primers used for PCR. RB, T-DNA right border; LB, T-DNA left border. **(B)** Semiquantitative RT-PCR on different regions of the *AtPolλ* gene. Primer pairs used for RT-PCR were [A Fw + A Rev], [B Fw + B Rev], [C Fw + C Rev], and [LBb1 + C Rev]. The Ribosomal S16 was used as a control. W, wild-type; M, *atpolλ-1* mutant. **(C)** Structure of *AtPolλ* cDNA in *atpolλ-1* mutants. Capitals, *AtPolλ* gene sequence; small letters in the box, pROK2 sequence; small letters/italics/bold, filler DNA; small letters/italics, intron sequence, capitals below a DNA sequence, amino acids; asterisk, stop codon. Each number represents the position of nucleobase in the *AtPolλ* cDNA sequence. **(D)** Structure of AtPolλ proteins in wild type vs *atpolλ-1* mutants. BRCT, BRCA1-C terminal domain; Pol X, DNA polymerase domain conserved among Pol X-family members.

We took a reverse genetic approach in order to examine the *in vivo* function of *AtPol λ*. The Salk T-DNA insertion collection was searched using the amino acid sequence of the rice Pol λ protein (GenBank Accession: BAD18976) as a template and the SALK_075391C line was found as a homozygous mutant carrying the T-DNA insertion in the *AtPol λ* gene (gene ID: At1g10520). Sequence analysis of the flanking regions of T-DNA revealed that the T-DNA was inserted in ninth intron of the *AtPol λ* gene (Figure 1A). To date, three *Arabidopsis polλ* mutants (*atpolλ-1, atpolλ-2*, and *atpolλ-3*) have been isolated from the SALK T-DNA insertion line (Roy et al., 2011). Because both the SALK_075391C mutant and the *atpolλ-1* mutant reported by Roy have the same allele, we designate this mutant as *atpolλ-1*. The effect of T-DNA insertion on transcription of *AtPol λ* in *atpolλ-1* mutants was investigated by RT-PCR. No amplification was obtained by RT-PCR with the polλ-1BF/ polλ-1BR primer combination, while other two primer combinations aiming to amplify either upstream or downstream region of the T-DNA insertion site produced RT-PCR products, suggesting that the homozygous mutant does not express a wild-type transcript (Figure 1B), as previously observed (Roy et al., 2011). Sequencing of the *AtPol λ* transcript in the mutant revealed that this transcript contains partial sequences of T-DNA and intron 9 and that the joint between the T-DNA's right border and intron 9 includes filler DNA (Figure 1C). Thus, this insertion event generates a new stop codon inside of the pol X domain of the AtPol λ protein, which, if translated, would result in the C-terminal truncation (Figure 1D). This predicted mutant AtPol λ protein would contain a BRCT domain in the N-terminal region. The pol X domain plays a critical role in DNA synthesis, while the BRCT domain interacts with other DNA repair proteins (Leung and Glover, 2011). Thus, we regarded *atpolλ-1* as a loss-of-function mutant lacking in a DNA synthesis capability but still retaining an ability to interact with other proteins. The *atpolλ-1* mutants are fertile and develop normally, and did not show any obvious phenotypic differences compared to with wild-type Col plants (data not shown).

### Sensitivity of the *atpol* λ-*1* mutant to various DNA damages

**Figure 2:**
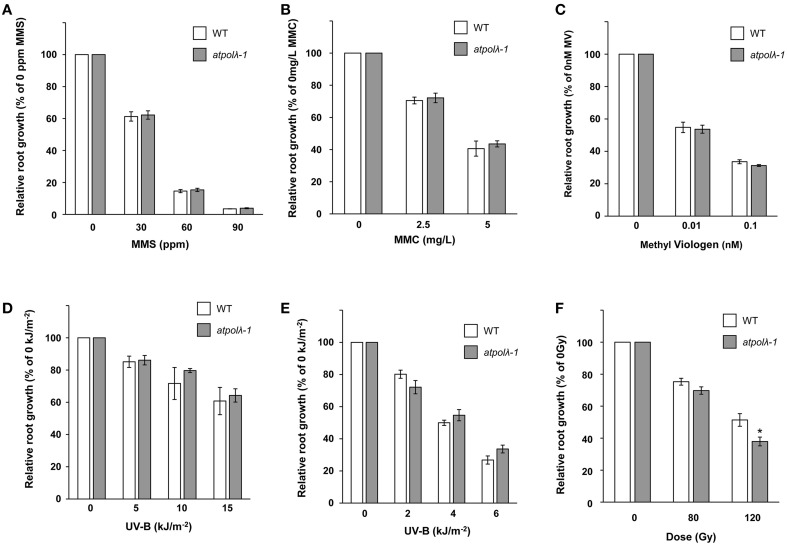
**Sensitivity of the** ***atpolλ-1*** **mutant to various DNA damages**. Sterilized wild-type and *atpolλ-1* seeds were sown on solid MS agar containing methylmethano sulfate **(A)** mitomycin C **(B)**, or methyl viologen **(C)**, and root length was measured after 7 days. The 3 day-old wild-type and *atpolλ-1* seedlings grown on MS plates were exposed to UV-B, and then grown for two more days under normal light **(D)** or orange light **(E)**. Sterilized wild-type and *atpolλ-1* seeds were γ-irradiated and sown on MS-agar plates. Root length was measured after 7 days **(F)**. Error bars represent the standard error of the mean of three independent experiments with 24–32 seedlings (average *n* = 30) for 2A, with 23–36 seedlings (average *n* = 28) for 2B, with 15–32 seedlings (average *n* = 25) for 2C, with 13–23 seedlings (average *n* = 20) for 2D, with 12–19 seedlings (average *n* = 15) for 2E, and with 18–48 seedlings (average *n* = 28) for 2F per line, per concentration, per replicate plates. The difference in relative root growth between wild type and *atpolλ-1* mutants was significant at 120 Gy in Panel **(F)** (^*^p < 0.05, *t*-test).

**Figure 3:**
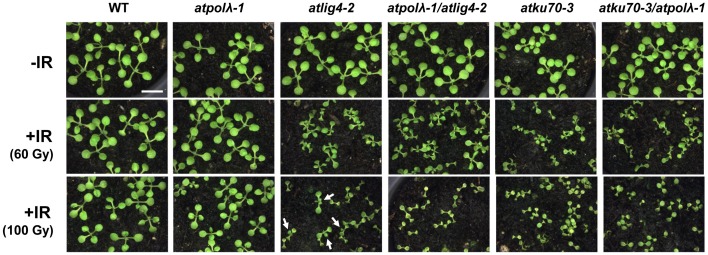
**Effects of γ-irradiation on true leave formation in wild-type and mutant plants**. Wild-type and mutant seeds were exposed to gamma-radiation at 0 (mock-irradiation) or 100 Gy, and sown on soil. These images were captured 10 days after γ-irradiation. Bar = 1 mm. All pictures were taken at the same magnification. Arrows show true leaves with abnormal shapes produced after γ-irradiation.

Previous studies have reported that mammalian *Pol λ* is involved in NHEJ and short-patch BER (Fan and Wu, 2004; Lee et al., 2004; Braithwaite et al., 2005a,b, 2010; Garcia-Diaz et al., 2005; Nick McElhinny et al., 2005). However, mice *Pol λ* deficient cells showed hypersensitivity to oxidative DNA damages but not to ionizing radiation (IR) (Kobayashi et al., 2002; Braithwaite et al., 2005b). This paradox might arise from the fact that mammals have four pol X family polymerases whose functions are partly overlapped. Higher plants are ideal organisms to study functions of *Pol λ* gene because *Pol λ* is the only member of the pol X-family in higher plants. In order to investigate its role in plant DNA repair, we first examined sensitivity of *atpolλ-1* mutants to various types of DNA damage by comparing root growth with or without DNA damaging treatments. The *atpolλ-1* mutants showed wild-type levels of sensitivity to methylmethane sulfonate (DNA alkylation), mitomycin C (cross-links), and methyl viologen (oxidative damage) when compared to wild-type plants (Figures 2A–C). UV radiation induces both cyclobutane pyrimidine dimers (CPDs) and 6-4 photoproducts (6-4 PPs) in DNA. These UV-B induced damages are repaired via blue-light-dependent photorepair catalyzed by photolyases and through light-independent nucleotide excision repair (NER). Growth of the light-treated *atpolλ-1* mutants after UV-B irradiation was similar to wild type, while the mutant grown under orange light showed a slightly increased, but not statistically significant, resistance at the dose of 6 kJm^−2^ (Figures 2D,E). The *atpolλ-1* mutants exhibited a mild but statistically significant sensitivity to IR. Irradiation of γ-ray at a dose of 120 Gy inhibited root growth of *atpolλ-1* mutants more effectively that of wild-type plants (*P* < 0.05, Figure 2F). Next, we tested the effects of γ-irradiation on formation of true leaves. The size of γ-irradiated seedlings was similar between wild-type and the *atpolλ-1* mutants (Figure 3), however the number of true leaves was decreased in the 100 Gy-irradiated *atpolλ-1* mutants (*P* < 0.05, Table 1). Gamma irradiation induces both DNA double strand breaks (DSBs) and oxidative damage. Given that we had not observed sensitivity to methyl viologen, this result suggests that *AtPol λ* may be involved in DSB repair.

**Table 1:** **The number of true leaves among DSB repair-deficient mutants 7 days after irradiation**.

**Genotype**	**The number of true leaves 7 days after irradiation (Average ± SD)**				
	**Dose (Gy)**				
	**0**	**40**	**60**	**80**	**100**
WT Col	2.00 ± 0.00 (55)	1.98 ± 0.03 (48)	2.00 ± 0.00 (46)	2.00 ± 0.00 (54)	1.97 ± 0.04 (43)
*atpolλ-1*	2.00 ± 0.00 (47)	1.96 ± 0.06 (55)	1.98 ± 0.02 (60)	1.79 ± 0.05 (65)	1.46 ± 0.08 (53)^*^
*atlig4-2*	2.00 ± 0.00 (40)	1.31 ± 0.14 (66)	0.29 ± 0.06 (58)^*^	0.05 ± 0.07 (70)^*^	0.02 ± 0.03 (70)^*^
*atpolλ-1*/*atlig4-2*	2.00 ± 0.00 (42)	0.83 ± 0.08 (61)^*^	0.23 ± 0.32 (73)	0.00 ± 0.00 (65)^**^	0.08 ± 0.11 (54)^*^
*atku70-3*	1.92 ± 0.03 (49)	0.75 ± 0.28 (55)	0.11 ± 0.15 (47)^*^	0.00 ± 0.00 (57)^**^	0.00 ± 0.00 (67)^*^
*atku70-3*/*atpolλ-1*	2.00 ± 0.00 (55)	0.57 ± 0.17 (70)^*^	0.03 ± 0.01 (63)^**^	0.03 ± 0.04 (62)^**^	0.00 ± 0.00 (58)^*^

*Seeds were imbedded in cold water for 2 days before irradiation. The γ-ray irradiated seeds from a ^137^Cs source were sown on soil and grown for 7 days in a growth chamber under cool-white lamps at an intensity of 100–150 μmol m^−2^ s^−1^ with a cycle of 16 h day/8 h night at 20°C. Values are mean ± standard deviation (SD). Numbers in parenthesis indicate the total number of plants scored across all two replicates*.

* Significant at P < 0.05;

** *significant at P < 0.01*.

### Genetic analysis of *Atpol* λ function in DSB repair

DSBs are repaired through both NHEJ and HR (homologous recombination) pathways. Ku heterodimer (Ku70 and Ku80) and Lig4 play a critical role in NHEJ in all eukaryotes, their homologs have been identified in *Arabidopsis* (Bundock et al., 2002; Riha et al., 2002; Tamura et al., 2002; Friesner and Britt, 2003), and *Arabidopsis* mutants defective in these genes are hypersensitive to IR. Our observations that the *atpolλ-1* mutants showed sensitivity to IR imply that Pol λ may be involved in the repair of IR-induced breaks in *Arabidopsis*. To elucidate the relation between *AtPol λ* and these NHEJ core genes in DSB repair, we made double knockout mutants by crossing *atpolλ-1* with either *atku70-3*, a newly isolated T-DNA mutant from the SALK T-DNA insertion collection in the Col background (Figure S1), or the *atlig4-2* mutant (Friesner and Britt, 2003). IR did not influence true leaf formation of wild-type plants, while the number of true leaves was decreased in the 80 Gy- and 100 Gy-irradiated *atpolλ-1* mutants (Table 1). Inhibition of true leaf formation clearly appeared in four mutants except the *atpolλ-1* mutants irradiated as seeds at 40 Gy radiation (Table 1). At this dose, the *atlig4-2* mutant was able to produce at least one true leaf on average while irradiated *atpolλ-1*/*atlig4-2, atku70-3*, and *atku70-3*/*atpolλ-1* mutants produced less than one true leaf. Seven days after 60 Gy or higher dose γ-irradiation, formation of true leaves was severely inhibited in *atlig4-2* and *atku70-3*, as previously observed. The double *atpolλ-1*/*atlig4-2* and *atku70-3*/*atpolλ-1* mutants were not significantly more sensitive to IR than the *atku70-3* and *atlig4-2* mutants, although they tended to show slightly higher sensitivity than each single mutant (Table 1). Phenotypic difference in true leaf formation among mutants appeared 10 days after 100 Gy dose irradiation. The wild-type and *atpolλ-1*mutants produced normal true leaves and some *atlig4-2* mutants were able to produce one or two true leaves with abnormal shapes. However, other three mutants had only cotyledons (Figure 3).

**Figure 4:**
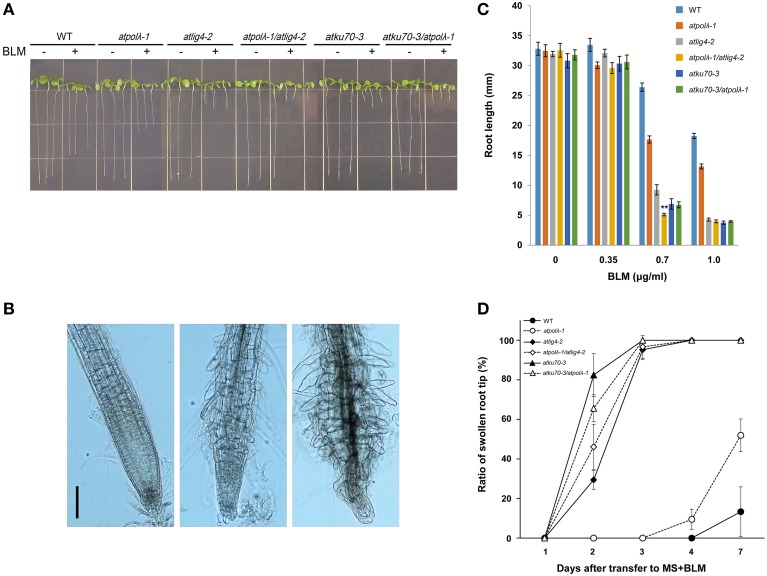
**Effects of bleomycin-induced DSBs on root growth. (A)** Root length of 1 week-old wild-type and mutant seedlings grown on MS agar containing 0.7 μg mL^−1^ bleomycin (BLM). **(B)** Morphology of BLM-treated mutant primary root tips. The 3 day-old seedlings were transferred to MS agar plates supplemented without (left panel, mock treatment) or with 0.1 μg ml mL^−1^ bleomycin (center and right panels). Images were captured 1 day after transfer. Abnormal root tips displayed disorganized root tip structure; randomly swelling cells and emerging of rounded root hairs above the meristem. Bar = 100 μm. **(C)** Root length of 1 week-old wild-type and mutant seedlings grown on MS plates containing 0, 0.35, 0.7, and 1.0 μg mL^−1^ BLM. Error bar represents the standard error of the mean of three independent experiments with 17–36 seedlings (average *n* = 29) per line, per concentration, per replicate plates. ^**^Significantly different from the value of the *atpolλ-1* and *atlig4-2* mutants (^**^P < 0.01). **(D)** Ratio of abnormal root tips after transplant to MS agar plates containing 0.1 μg ml mL^−1^ BLM. Error bar represents the standard error of the mean of three independent experiments with 20–23 seedlings (average *n* = 21) per line, per concentration, per replicate plates.

Sensitivity of these mutants to DSBs was also analyzed by investigating the effects of bleomycin (BLM), a radiomimetic reagent that generates DSBs, on root growth (Figures 4A–D). Roots of mutants exposed to BLM exhibited morphological changes such as short root length, swollen root tips, and disorganized layers (Figures 4A,B). Root length of treated plants was similar to those of untreated control plants at 0.35 μg mL^−1^ of BLM. Inhibition of root growth became obvious at 0.7 μg mL^−1^ BLM. The *atpolλ-1* mutants were more sensitive than wild-type plants, but exhibited mild sensitivity when compared to other four mutants. Sensitivity of the *atpolλ-1*/*atlig4-2* double mutants was higher than each *atpolλ-1* and *atlig4-2* single mutant (*P* < 0.05). On the other hand, no significant difference was observed between *atku70-3* vs. *atku70-3*/*atpolλ-1* mutants. The 1.0 μg mL^−1^ BLM-treated wild type and mutant roots showed a similar inhibition tendency as seen in the 0.7 μg mL^−1^ BLM-treated plants although root length became shorter than that of 0.7 μg mL^−1^ BLM treated-plants (Figure 4C). Next, we examined the timing when swollen root tips appeared after BLM treatment. At 0.1 μg mL^−1^ of BLM, swelling of root tips occurred 2 days after transplant in *atlig4-2, atpolλ-1*/*atlig4-2, atku70-3, atku70-3*/*atpolλ-1* mutants although the ratio of abnormal root tip shape differed among four mutants. The *atku70-3* mutants were more sensitive than *atlig4-2* mutants despite the fact that both genes play critical roles in the canonical NHEJ pathway. Both the *atpolλ-1* mutants and wild type root tips shape appeared to be normal until 3 days after transplant, but became abnormal in the *atpolλ-1* mutants 4 days and in the wild-type plants 7 days after transplant, respectively (Figure 4D). A similar sensitivity pattern among wild-type and mutants was observed even at 0.25 μg mL^−1^ BLM although the ratio of abnormal root tip in wild-type and the *atpolλ-1* mutants was higher (Table S2).

Taken together, the IR- and BLM-sensitivity indicate that *AtPol λ* has a function in DSB repair in plants. Ku complex and DNA ligase 4 have already been implicated in the canonical NHEJ pathway. It is entirely possible that AtPol λ also participates in this Ku/Lig4 pathway of NHEJ, but if so, it is not essential for every repair event catalyzed by these enzymes, as the *atpolλ-1* mutant clearly does not share the hypersensitivity of *atku70-3* and *atlig4-2* to higher doses of BLM and IR.

### DNA damage response in mutants

**Figure 5:**
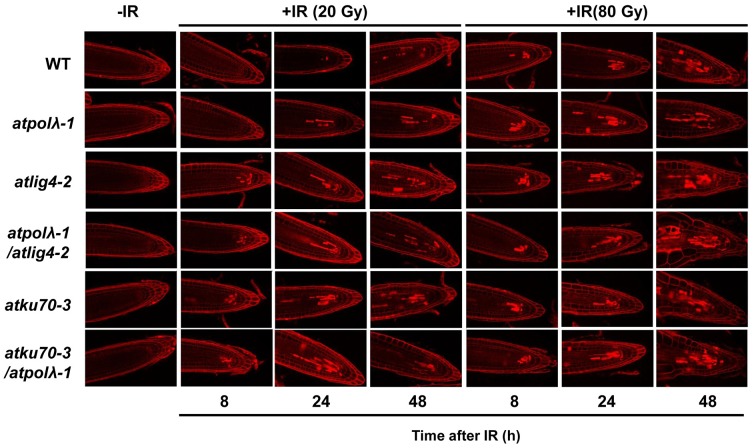
**Programmed cell death in wild-type and mutants after IR**. The 5 day-old seedlings in each mutant background were exposed to 20 Gy or 80 Gy. Seedling before irradiation were used as control. The γ-irradiated seedlings were collected at 8, 24, and 48 h after irradiation and dead cells in root tips were visualized by PI-staining. Bar = 50 μm.

Previous studies demonstrate that DSBs trigger two robust responses in plants: programmed cell death (PCD) and the ATM/ATR/SOG1-dependent expression of an enormous number of genes (Culligan et al., 2004, 2006; Fulcher and Sablowski, 2009; Yoshiyama et al., 2009; Furukawa et al., 2010). This PCD requires ATM or ATR, and the SOG1 transcription factor and is largely restricted to a specific subset of the cells of the root tip meristem- the precursors of the stele. It is possible that PCD occurs with higher frequency in γ-irradiated mutant root tips that are deficient in DNA repair. To test this hypothesis, we examined post-irradiation (20 Gy and 80 Gy) PCD events in 5 day-old seedlings of mutant and wild-type plants (Figure 5 and Figure S2). In 20 Gy-irradiated wild-type plants, PCD first occurred sometime between 8 and 24 h after radiation and the frequency of dead cells was decreasing by 72 h after radiation (Figure S2). The 20 Gy-irradiated *atpolλ-1* mutants showed a similar cell death pattern to wild-type plants, with perhaps a slight enhancement in the frequency and persistence of dead cells. In contrast, in the other four mutants dead cells were observed by 8 h after 20 Gy radiation, the PI-staining was more persistent. Moreover, initiation of swelling of root tips was observed in the *atku70-3* mutants at 72 h after radiation (Figure S2). Gamma-irradiation at 80 Gy induced more PCD events in both wild-type and mutants at 8 h after radiation (Figure 5). Enlargement of root tip cells occurred in four mutants (*atlig4-2, atpolλ-1*/*atlig4-2, atku70-3*, and *atku70-3*/*atpolλ-1*), while wild-type and the *atpolλ-1* root tips displayed slightly swollen but still kept normal root tip shape. These results suggest that AtPol λ is involved in resistance to IR-induced meristematic death, but is not as critical to this process as AtKu70 or AtLig4.

**Figure 6:**
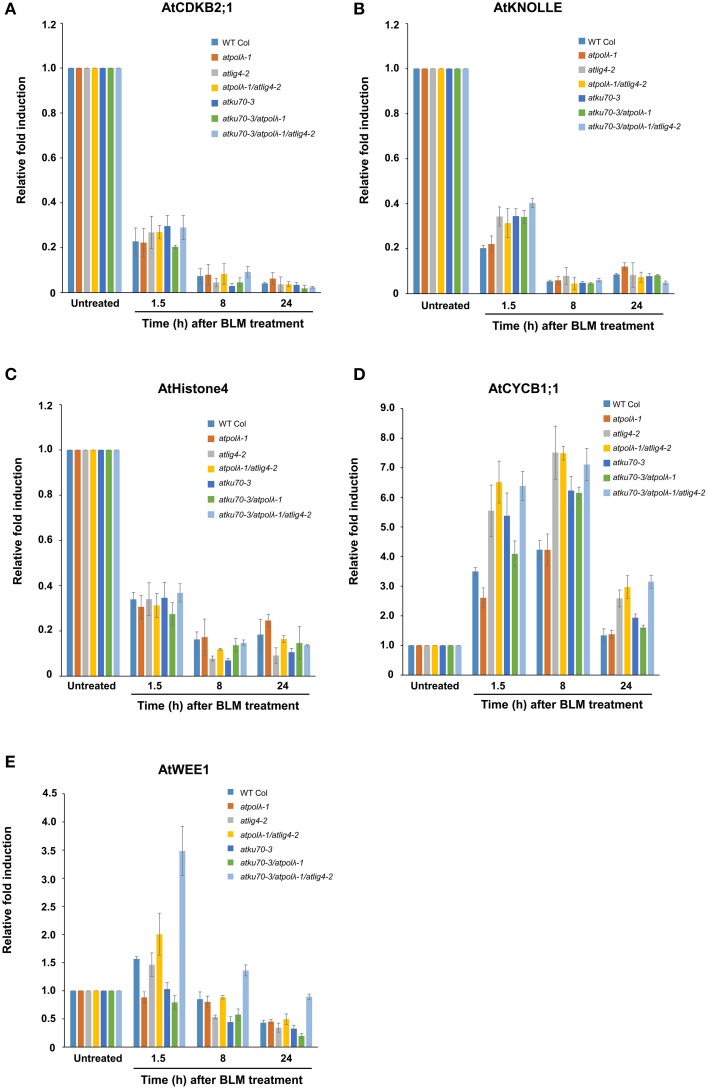
**Transcriptional responses of cell cycle-related genes in wild-type and mutants in response to DSBs**. Expression levels of **(A)** *AtCDKB2;1*, **(B)** *AtKNOLLE*, **(C)** *AtHistone H4*, **(D)** *AtCYCB1;1*, and **(E)** *AtWee1* after BLM treatment. Five day-old wild-type and mutant seedlings grown on MS agar plates were embedded in water containing 30 μg mL^−1^ BLM for 1.5 h. Followed by rinse with sterilized water, BLM-treated seedlings were transferred onto MS agar plates before collection at each time point after treatment. All values were normalized to the expression level of control genes. Error bars indicate the standard error of the mean.

Next, we performed quantitative RT-PCR (qRT-PCR) to investigate whether lack of NHEJ influences transcriptional responses to IR (Figures 6A–E). Expression of three cell cycle marker genes and two cell cycle checkpoint genes was measured up to 24 h after 30 μg mL^−1^ BLM treatment. *AtCDKB2;1* (Figure 6A, G2 phase marker), *AtKNOLLE* (Figure 6B, M phase marker), and *AtHistone4* (Figure 6C, S phase marker) were selected as each phase-specific marker gene. Our qRT-PCR analysis showed that expressions of all marker genes were significantly downregulated at 1.5 h after BLM treatment and had not recovered by 24 h after treatment (*P* < 0.05 or *P* < 0.01). We used *AtCYCB1;1* and *AtWee1* as a marker of cell cycle checkpoint genes (Figures 6D,E). *AtCYCB1;1* is a plant-specific B-type cyclin playing an unique role in DDR pathway, and DSB-inducible accumulation of *AtCYCB1;1* transcripts reflects G2/M cell cycle arrest (Culligan et al., 2006). *AtWee1* is a protein kinase controlling the progression of plant cell cycle in an ATM/ATR-dependent manner; lack of *AtWee1* causes extension of S-phase as well as more PCD events in response to replication stresses (Sorrell et al., 2002; De Schutter et al., 2007; Cools et al., 2011). Rapid upregulation of *AtCYCB1;1* occurred in all genotypes at 1.5 h after BLM treatment although the degree of expression level varied among mutants. The induction of *AtCYCB1;1* expression continued at 8 h after treatment, and DSB-inducible upregulation of *AtCYCB1;1* observed at 1.5 and 8 h after treatment in wild-type and all mutants was significant (*P* < 0.05 or *P* < 0.01). At 24 h after treatment the expression of *AtCYCB1;1* in wild-type and *atpolλ-1* mutants was recovered to the untreated level, whereas it still remained significantly high level in other five mutants (*P* < 0.05 in *atku70-3* and *atku70-3*/*atpolλ-1* mutants and *P* < 0.01 in *atlig4-2, atpolλ-1*/*atlig4-2*, and *atku70-3*/*atpolλ-1*/*atlig4-2* mutants). As shown in Figure 6E, an induced expression of *AtWee1* occurred in wild-type and the *atpolλ-1*/*atlig4-2*/*atku70* triple mutant at 1.5 h after treatment (*P* < 0.01). At later time point *AtWee1* expression gradually decreased. The *AtWee1* expression in wild-type recovered to the untreated level, but high expression of *AtWee1* continued in the triple mutant at 8 h after treatment (*P* < 0.05). Except the triple mutant, the *AtWee1* expression was significantly decreased in BLM treated plants at 24 h after treatment (*P* < 0.05 or *P* < 0.01). Taken together, these expression data suggest that DSB-inducible G2/M cell cycle arrest equally occurs in both wild-type and six mutants and that DSBs may prolong the duration of S-phase of the *atku70-3*/*atpolλ-1*/*atlig4-2* triple mutant.

**Figure 7:**
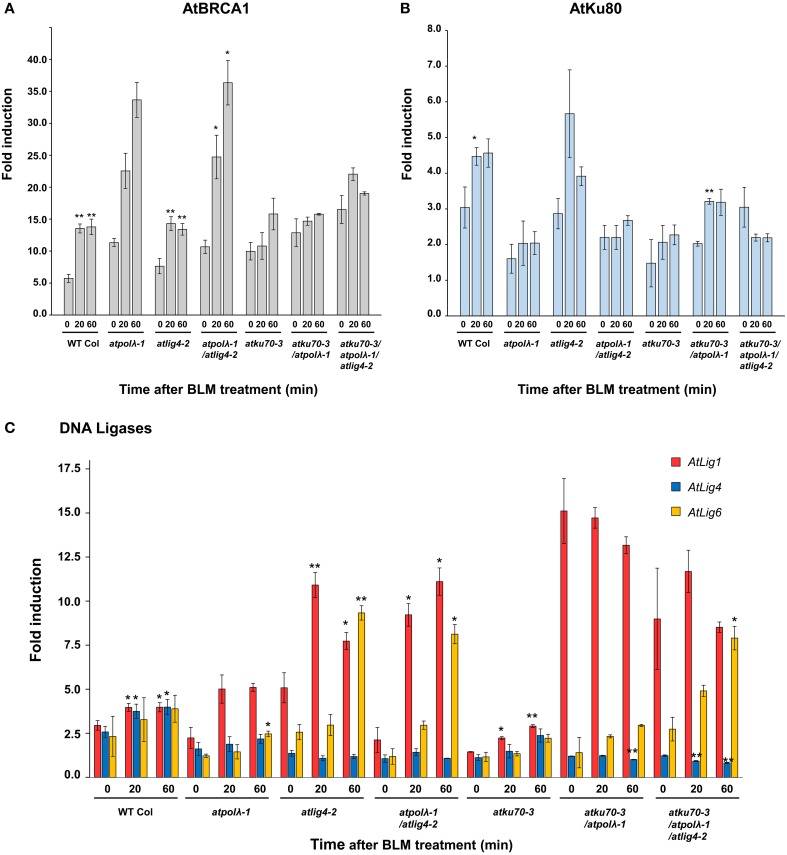
**Transcriptional responses of DSB repair genes in wild-type and mutants in response to DSBs**. Expression levels of **(A)** *AtBRCA1*, **(B)** *AtKu80*, and **(C)** DNA ligases (*AtLig1, AtLig4*, and *AtLig6*) after BLM treatment. Five day-old wild-type and mutant seedlings grown on MS agar plates were embedded in water containing 30 μg mL^−1^ BLM for 1 h. Followed by rinse with sterilized water, BLM-treated seedlings were transferred onto MS agar plates before collection at each time point after treatment. All values were normalized to the expression level of control genes, and then relative expressions compared to untreated controls were calculated. Error bars indicate the standard error of the mean. ^*^Significant at *P* < 0.05; ^**^significant at *P* < 0.01.

It is possible that lack of *AtPol λ* and other NHEJ-involved genes may induce expression of substitute DNA repair genes to compensate for lost functions. To test this hypothesis, expressions of *Arabidopsis BRCA1* (as a positive control for induction), *Ku80*, and three DNA ligases (*Lig1, Lig4*, and *Lig6*) were measured by qRT-PCR immediately after BLM treatment (*t* = 0) and after 20 and 60 min of repair recovery (Figures 7A–C). *BRCA1*, breast cancer susceptibility gene 1, is a signal transducer largely linked to the ATM pathway required for the efficient repair of DSBs by homologous recombination in somatic cells of *A. thaliana* with strongly induced transcription by IR (Lafarge and Montane, 2003). DSBs generated by BLM treatment strongly upregulated *AtBRCA1* expression in wild-type and mutants (Figure 7A). *AtBRCA1* expression in the *atpolλ-1* mutant was dramatically enhanced from 10- at *t* = 0 to 33-fold after 60 min of repair, and even higher induction from 10- to 40-fold was observed in the *atpolλ-1/atlig4-2* double mutants. In all other lines, the expression of *AtBRCA1* during repair period was only slightly higher than that of wild-type and ranged from 10- to 20-fold. In contrast to *AtBRCA1*, the expression of *AtKu80* was not significantly induced after BLM treatment. *AtKu80* expression resulted in 2- to 6-fold increase compared to untreated controls during the recovery period (Figure 7B). Except the *atku70-3*/*atpolλ-1*/*atlig4-2* mutant *AtKu80* expression tended to increase as the recovery time went. *AtKu80* expression was significantly induced in wild-type and the *atku70-3*/*atpolλ-1* mutants at 20 min after treatment (*P* < 0.05 or *P* < 0.01, Figure 7B). Figure 7C showed DSB-induced upregulation of three DNA ligase genes in wild-type and mutants. In wild-type, all assayed ligases were slightly induced (2- to 3-fold) compared to untreated control, and they showed a similar expression pattern during repair period. At 20 and 60 min after BLM treatment *AtLig1* and *AtLig4* expressions were different from expressions at 0 min after treatment (*P* < 0.05). An induction pattern of ligase genes among all mutant lines differed from wild-type. In the *atpolλ-1* mutant *AtLig1* and *AtLig6* expressions were induced during the repair period, but no induction of *AtLig4* occurred. The expression of *AtLIG1* increased from 2.2-to 5.1-fold during the repair period, and *AtLig6* expression showed 2.5-fold increase at 60 min after treatment. Compared to wild-type, *AtLig1* expression was strongly induced in mutants whose genetic background was *atlig4-2* (*atlig4-2, atpolλ-1*/*atlig4-2*, and *atku70-3*/*atpolλ-1*/*atlig4-2*). The strong induction of *AtLig1* expression was also observed in the *atpolλ-1*/*atku70-3* mutants. It reached up to 15-fold increase at 0 min after treatment and then constantly kept a high level of expression even 60 min after treatment. Interestingly, the expression of *AtLig6* was gradually increased 3- to 10-fold in all *AtLig4* mutated lines during recovery period (*P* < 0.05 or *P* < 0.01), while *AtLig6* expression was not significantly induced in the *atku70-3*/*atpolλ-1* mutant. In the *atku70-3* mutants, only *AtLig1* expression was slightly induced after treatment although its expression level was lower than wild-type.

### Lack of *atpol* λ and other NHEJ-involved genes affects transformation efficiency

**Figure 8:**
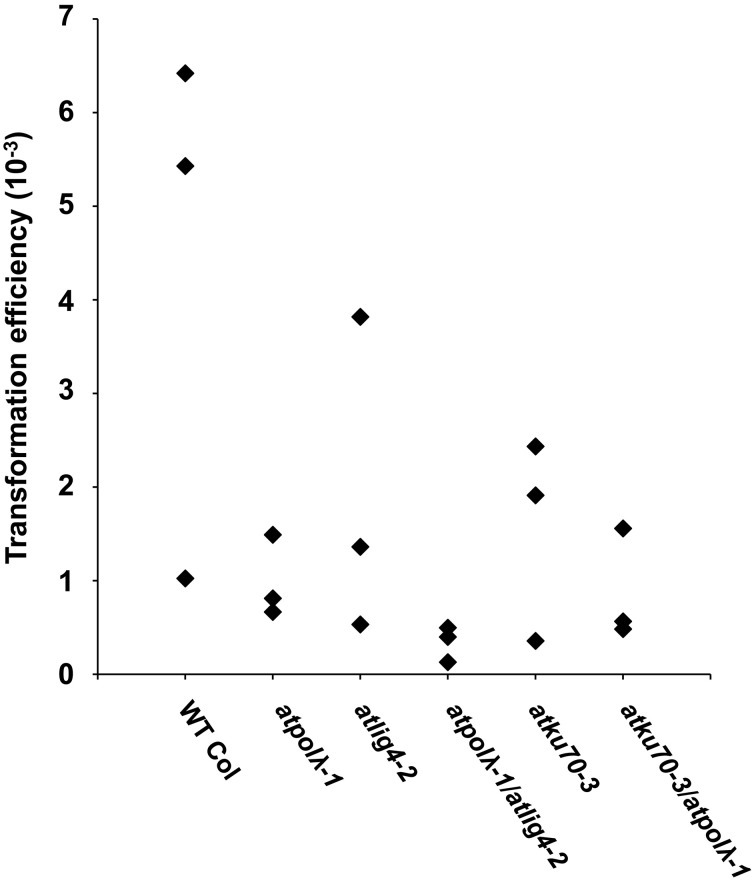
**Transformation efficiency in wild-type and DSB-deficient mutants**. The pFLAR101 vector was transformed into T_0_ plants through *Agrobacterium*-mediated floral dipping and T_1_ seeds were harvested as described in Materials and Methods. The T_1_ transformed seeds overexpressing the DsRED protein were selected by fluorescence microscopic observation and transformation efficiency was calculated based on the number of fluorescent seeds. Each rhombus represents transformation efficiency per line per trial. Transformation experiments were repeated three times.

It has been reported that DSB repair plays a critical role in integration of transgenes in plants (Friesner and Britt, 2003; Li et al., 2005; Mestiri et al., 2014). In order to uncover functions of these DSB repair genes in this process, we investigated their transformation efficiency using the pFLUAR101 reporter construct. This pFLUAR101 reporter construct contains both the promoter for the seed storage protein napin driving the DsRED gene. Transformed embryos display red fluorescence due to accumulation of the DsRED protein. This enables us to identify transformed seeds using a fluorescent microscope (Stuitje et al., 2003). The pFLUAR101 construct was transformed into wild-type and five mutants (*atpolλ-1, atlig4-2, atpolλ-1*/*atlig4-2, atku70-3*, and *atku70-3*/*atpolλ-1*) by *Agrobacterium*-mediated floral dip, and then the transformation efficiency of each plant line was calculated based on the number of T_1_ DsRED seeds (Figure 8 and Table S3). All DSB-deficient mutants tended to show a lower transformation efficiency than wild-type plants in each trial despite the fact that it was not statistically significant due to a wide range of transformation efficiencies over three trials (Figure 8). As shown in Table S3, the *atpolλ-1* mutants showed a lower transformation efficiency than wild-type, and tended to exhibit lower efficiency than *atlig4-2* and *atku70-3* mutants. The reduction of transformation efficiency of the *atpolλ-1* was 1.5- to 8-fold compared to wild-type, 1.7- to 3.0-fold for *atlig4-2*, and 1.3- to 3.0-fold for *atku70-3*, respectively. The *atpolλ-1*/*atlig4-2* double mutants showed a lower transformation efficiency than *atpolλ-1* or *atlig4-2* single mutant and its transformation efficiency in each trial was the lowest among five mutants (Table S3). In contrast, no significant difference was observed among *atku70-3, atpolλ-1*, and *atku70-3*/*atpolλ-1* mutants (Figure 8). These results suggest that *AtPol λ* may play a more important role in transgene integration than either *AtLig4* or *AtKu70*.

## Discussion

Despite the fact that only Pol λ is encoded in plant genomes among Pol-X family members, its biological function is poorly understood. In this paper, we took a reverse genetic approach to study Pol λ functions in various plant DNA repair pathways. The expression of Pol λ is induced by MMS treatment, MMC treatment, and UV-B radiation, suggesting that plant Pol λ may participate in repair of alkylated DNA, DNA crosslink, and UV-damaged DNA (Uchiyama et al., 2004; Roy et al., 2011, 2013). However, the *atpolλ-1* mutant exhibits sensitivity to DSBs caused by IR or bleomycin treatment, but does not display hypersensitivity to other DNA damages such as DNA alkylation (MMS), crosslink (MMC), oxidative damages (MV), and UV damages such as CPDs and 6-4 PPs (UV-B). Analysis of the *AtPol λ* transcript in the *atpolλ-1* mutant reveals that the transcriptional error occurs in this mutant, which results in producing the truncated Pol λ protein (Figure 1D). The intact AtPol λ protein consists of two major domains; N-terminus and Pol β-like C-terminus (Roy et al., 2011). The N-terminus, which is comprised of 198 amino acid residues (aa), includes a nuclear localization signal (13 aa), a BRCT domain (96 aa), and a Ser-Pro-rich domain (91 aa). The C-terminus domain is occupied by the Pol X domain (329 aa) consisting of 8 kDa domain as well as fingers, palm and the thumb subdomains. Insertion of T-DNA of the *atpolλ-1* occurs in in intron within the palm subdomain of the Pol X domain. The loss of half of the palm, the entire thumb, and several catalytic residues (the equivalent of the human Pol λ amino acids R488 and E529, as described in Cisneros et al. (2008) and aligned in Roy et al., 2013) indicates that this truncated protein lacks any significant DNA polymerase activity. In contrast, the mutant protein still contains the intact 8 kDa and BRCT domains and might possess dRP lyase activity if the mutant protein is stable. In an earlier study (Roy et al., 2013), Roy et al. analyzed three different alleles (including *atpolλ-1*), and found no sensitivity to MMS. The *atpolλ-2* and *atpolλ-3* mutants carry insertions in the 5′ UTR and the last exon, respectively, and thus might conceivably express an functional protein, however, the authors were unable to detect Pol λ via Western blot and therefore the level of this protein of wild-type size in these two mutants, must be quite low. Thus, we propose two possibilities to explain the lack of sensitivity of the *atpolλ-1* mutants to MMS treatment; (1) the truncated Pol λ protein may play a role in repair of alkylated and oxidative DNA damages via preserved dRP lyase activity (though this would require that *atpolλ-2* and *atpolλ-3* are also expressing a functional protein, though none was detected via Western blot). (2) Plants prefer to use a *Pol λ*-independent repair pathway such as long-patch BER to repair AP sites generated by base repair glycosylases (we regard this as the simpler and more likely hypothesis).

In our hands the *atpolλ-1* mutant did not display UV-B sensitivity when irradiated as a seedling and then cultivated under non-photoreactvating light. We used the *Arabidopsis XPF* (*atxpf-2*) mutant as a control in our root-bending assay under dark condition because it was hypersensitive to UV-B radiation due to lack of NER (Jiang et al., 1997; Fidantsef et al., 2000). The relative root growth of UV-B irradiated *atxpf-2* mutants was decreased to 24% at a dose of 2 kJ m^−2^ and to 6% at 4 kJ m^−2^ compared to unirradiated control plants (unpublished data), which indicates that our UV-B treatment produces UV damages that sufficiently inhibit root growth of NER-deficient mutants. In contrast to our observation, the *atpolλ-1* mutant showed hypersensitivity when seeds were exposed to UV-B and seedlings germinated from UV-B radiated seeds had slower repair rates for both CPDs and DSBs (Roy et al., 2011). The 5 day-old *atpolλ-1* mutant seedlings were radiated with UV-B for a short period (ex. 18 s radiation for 1 kJ m^−2^) in our experiment, whereas mutant seeds are irradiated for 60 min at the dose of 5.4 kJ m^−2^ before sowing in Roy's experiment. About sensitivity to MMC, the sensitive phenotype of the *atpolλ-1* mutants is observed only when mutant plants are grown on MS agar plates supplemented with 10 μg ml^−1^ MMC, and the phenotypic difference between wild-type plants and the *atpolλ-1* mutants is not statistically significant at 3 and 5 μg ml^−1^ MMC (Roy et al., 2013). Taken these findings together, it is possible that the effect of Pol λ on repair of UV-B induced damage or DNA crosslink is too subtle to detect in our growth assay and that bombardment of high-dose UV radiation for a long period or MMC treatment of mutant plants at higher concentration may be necessary to cause hypersensitive phenotype. Besides CPDs and 6-4 PPs, it is also known that UV-B radiation often induces reactive oxygen species that cause oxidative damages to DNA. Accumulation of unrepaired single strand breaks is often converted to DSBs if positions of breaks in the genome are very close. Interstrand crosslinks (ICLs) generated by MMC at replication forks stall the process of DNA replication, and then the collapse of ICL-stalled replication forks provokes DSBs. In this paper we have demonstrated by IR and radiomimetic chemical treatment that the *atpolλ-1* mutants display mild sensitivity to DSBs. Therefore, it could be also possible that hypersensitivity of *atpolλ-1* mutants to UV-B and MMC may reflect sensitivity to DSBs as well as direct UV damages (CPDs and 6-4 PPs) or ICLs.

As in animals, DSBs are thought to be mainly repaired by Ku- and Lig4-dependent NHEJ in plants (West et al., 2000, 2002; Bundock et al., 2002; Riha et al., 2002; Friesner and Britt, 2003; Gallego et al., 2003). Both *atku70* and *atlig4-2* mutants show hypersensitivity to DSBs generated by gamma-irradiation or BLM treatment. Unlike these NHEJ-deficient mutants, the sensitivity of the *atpolλ-1* mutants is only observed when mutants are gamma-irradiated at high dose (100 Gy and 120 Gy) or treated with a high concentration of BLM (0.7 μg mL^−1^ and 1.0 μg mL^−1^). Moreover, the fraction of root tips with disorganized structure in the *atpolλ-1* mutants is lower than that of *atku70* or *atlig4-2* mutant. The observation that the *atpolλ-1* mutant exhibits a mild sensitivity to IR suggests that AtPol λ participates in DSB repair, just as it participates in DSB repair in mammals. Results using double mutants provide additional hints to consider Pol λ functions in NHEJ. The *atpolλ-1*/*atlig4-2* double mutant always shows higher sensitivity than each single mutant, while the sensitivity of the *atku70*/*atpolλ-1* double displays similar sensitivity to the *atku70* mutant. In canonical NHEJ, broken ends of DNA strands are first shielded by the Ku70/Ku80 heterodimer and then the Lig4/XRCC4 complex ligates guarded DNA ends. Although AtKu70 and AtLig4 functions in the same NHEJ pathway, we found that the *atku70* mutant is more sensitive to DSBs than the *atlig4-2* mutant. Taken together, these results suggest that plants may have two pathways for DSB repair, AtLig4-dependent canonical (C-NHEJ) and AtLig4-independent alternative (A-NHEJ), pathways downstream of the DNA protection process catalyzed by AtKu70/AtKu80. Our results also suggest that AtPol *λ* is employed in A-NHEJ. Recent studies on mammalian DSB repair have shown that microhomology-mediated end joining (MMEJ) is one of backup NHEJ pathways in which Ku80 and poly(ADP-ribose) polymerases (PARP) play essential roles. *In vitro* studies show that human DNA polymerase λ and Lig1, but not Lig4, are required for sufficient MMEJ reaction (Liang et al., 2008; Crespan et al., 2012). Similar to mammals, a study using RNAi-silenced *AtLig1* demonstrates that *AtLig1* plays an important role in DSB repair as well as single strand break repair (Waterworth et al., 2009). In addition, our qRT-PCR analysis reveals that DSBs induce expression of *AtLig1* and *AtLig6* in mutants lacking *AtLig4*. Given that Ku80 and PARP-dependent MMEJ is conserved in plants (Jia et al., 2013), our data are consistent with a model in which *AtPol λ* functions in some A-NHEJ pathway, (possibly MMEJ), in concert with *AtLig1* and/or *AtLig6*.

Recent studies have demonstrated that plants have a robust DNA damage response to DSBs (Culligan et al., 2006; Ricaud et al., 2007; Yoshiyama et al., 2009; Furukawa et al., 2010; Missirian et al., 2014). To investigate the role of *AtPol λ* in DDR, we investigated both the frequency of PCD and the transcriptional response after BLM treatment. Although the number of dead cells is slightly increased in the *atpolλ-1* mutant compared to wild-type, its influence on frequency of PCD is smaller than NHEJ-defective mutants. The DSB-inducible transcriptional response appears to be similar between wild-type and mutants. BLM treatment causes upregulation of *AtCYCB1;1* expression, while it downregulates expressions of other cell cycle specific marker genes, *AtCDKB2;1, AtKNOLLE*, and *AtHistone H4*. These results suggest that cell cycle is arrested at G2/M in BLM-treated root tip cells although there is no direct evidence. The expression of *AtCYCB1;1* remains high level in *atlig4-2* mutants at 24 h after treatment. Given that the high expression of *AtCYCB1;1* is associated with the existence of unrepaired DSBs, this result may reflect that A-NHEJ requires more time to repair DSBs than the AtLig4-dependent pathway does. Moreover, *AtWee1* expression is highly induced only in the *atku70*/*atpolλ-1*/*atlig4-2* triple mutant and this high expression continues at 24 h after treatment. It has been reported that *AtWee1* controls many aspects of response to replication blocks (Cools et al., 2011). This result raises the possibility that response to DNA damage of the triple mutant is enhanced because its DSB repair activity via both C-NHEJ and A-NHEJ is completely lost.

It has been reported that NHEJ plays an important role in the integration of a transgene. The efficiency of T-DNA insertion to the plant genome is decreased in *atku80* and *atlig4* mutants though this effect is not consistently observed (Friesner and Britt, 2003; Li et al., 2005). Given that AtPol λ functions in A-NHEJ, the efficiency of T-DNA insertion in the *atpolλ-1* mutant is expected to be decreased as in NHEJ-defective mutants. Decreased T-DNA insertion efficiency is observed in the *atpolλ-1* mutant as anticipated. However, its frequency tends to be lower than that of *atku70* or *atlig4-2* single mutant. The *atpolλ-1*/*atlig4-2* double mutant shows the lowest transformation efficiency among six tested plant lines. These results suggest that T-DNAs may insert via either C-NHEJ or A-NHEJ.

In summary, the results and discussion presented here provide new insights into functions of AtPol λ in plant DSB repair. Although AtPol λ is suggested to participate in A-NHEJ, much remains unclear about its molecular machinery. Further studies would be required to clarify the role of *AtPol λ* in A-NHEJ.

### Conflict of interest statement

The authors declare that the research was conducted in the absence of any commercial or financial relationships that could be construed as a potential conflict of interest.

## Supplementary material

Supplementary Figure 1 **Structure of *Ku70* gene in the *atku70-3* mutant. (A)** Schematic structure of the *AtKu70* gene and its T-DNA insertion. Arrowheads represent gene-specific primers used for PCR. RB, T-DNA right border; LB, T-DNA left border. **(B)** Semiquantitative RT-PCR on different regions of the *AtKu70* gene. Primer pairs used for RT-PCR were LBb1 + Ku70R (left panel) and Ku70F + Ku70R (right panel). W, wild type; M, *atku70* mutant. **(C)** Sequence data for T-DNA: DNA junctions in the *atku70* mutant. Capitals, *AtKu70* gene sequence; Capitals/bold in the box, pROK2 sequence.

Supplementary Figure 2 **PCD and phenotypical differences of WT and mutants after γ-irradiation**. The 5-day-old seedlings in each mutant background were exposed to 20 Gy or mock-irradiated (0 h). The γ-irradiated seedlings were collected at 72 h after irradiation and dead cells in root tips were visualized by PI-staining. Bar = 50 μm.
