# Supplementary figures and images for: Arabidopsis DNA polymerase lambda mutant is mildly sensitive to DNA double strand breaks but defective in integration of a transgene
Source: Front Plant Sci. 2015 May 27;6:357. doi: 10.3389/fpls.2015.00357 (PMC4444747; doi:10.3389/fpls.2015.00357)

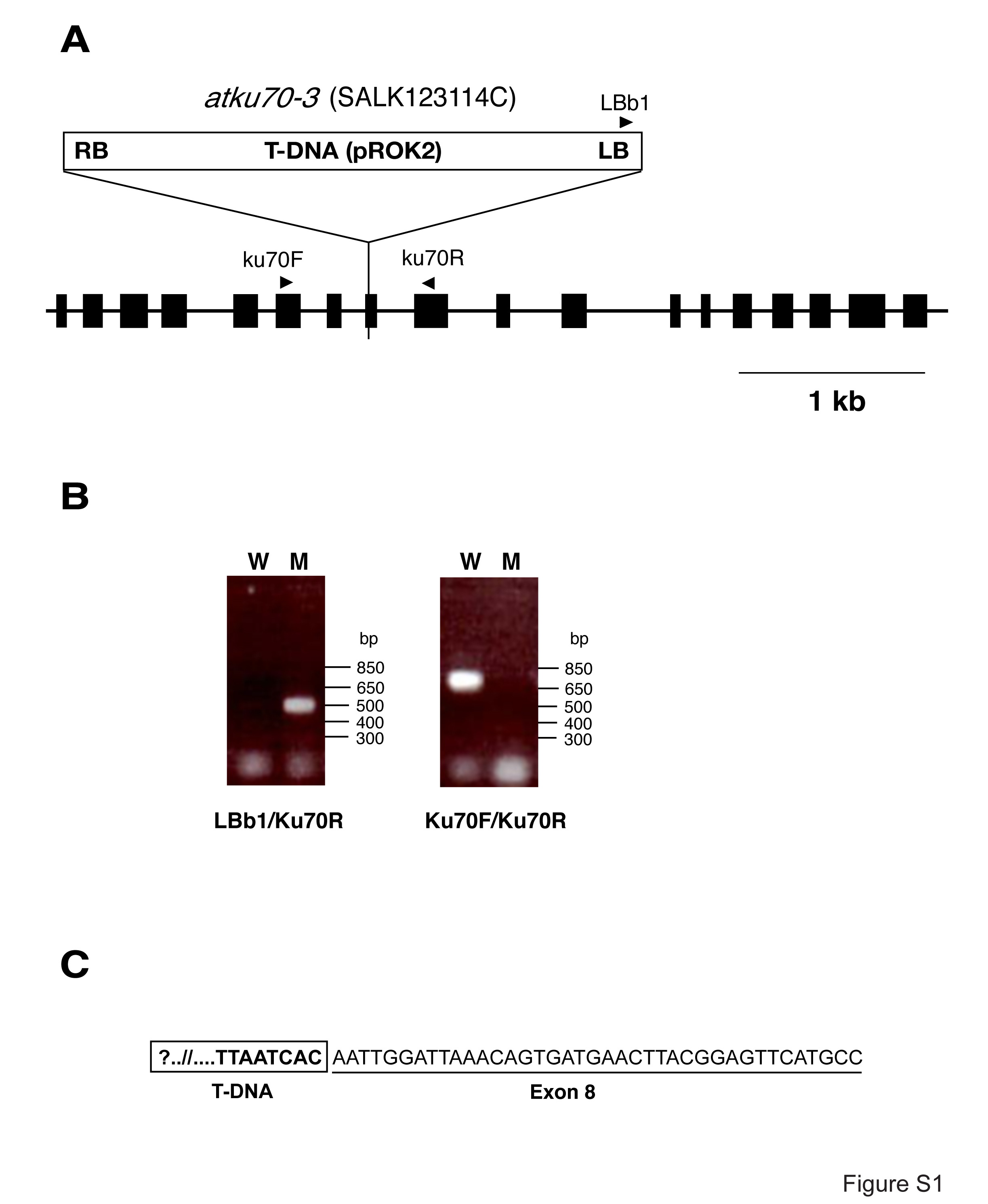

Supplement: Supplementary Figure 1 — Structure of Ku70 gene in the atku70-3 mutant. (A) Schematic structure of the AtKu70 gene and its T-DNA insertion. Arrowheads represent gene-specific primers used for PCR. RB, T-DNA right border; LB, T-DNA left border. (B) Semiquantitative RT-PCR on different regions of the AtKu70 gene. Primer pairs used for RT-PCR were LBb1 + Ku70R (left panel) and Ku70F + Ku70R (right panel). W, wild type; M, atku70 mutant. (C) Sequence data for T-DNA: DNA junctions in the atku70 mutant. Capitals, AtKu70 gene sequence; Capitals/bold in the box, pROK2 sequence. [file Image1.JPEG]

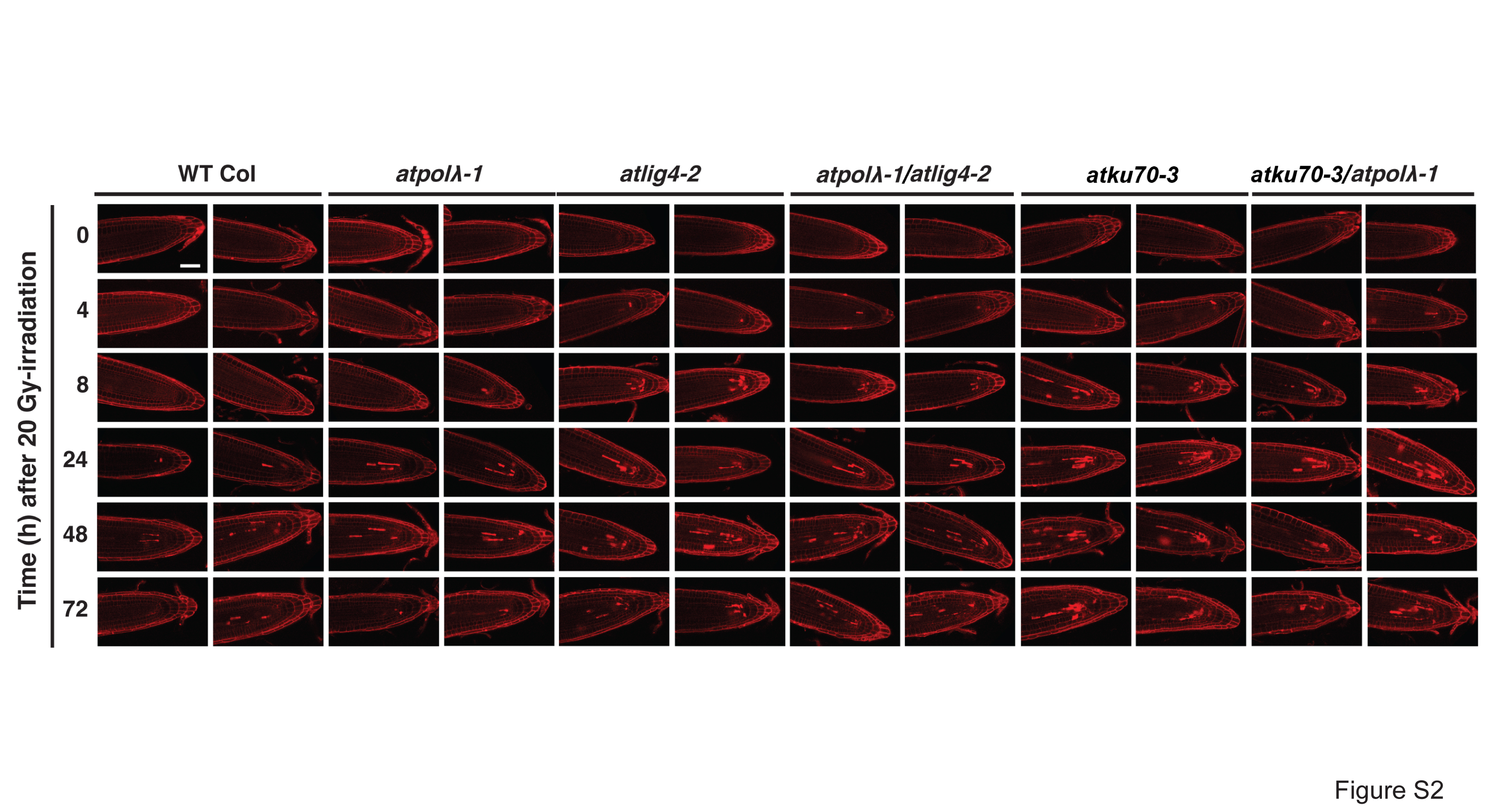

Supplement: Supplementary Figure 2 — PCD and phenotypical differences of WT and mutants after γ-irradiation. The 5-day-old seedlings in each mutant background were exposed to 20 Gy or mock-irradiated (0 h). The γ-irradiated seedlings were collected at 72 h after irradiation and dead cells in root tips were visualized by PI-staining. Bar = 50 μm. [file Image2.JPEG]
