# Supplementary material for: Arabidopsis DNA polymerase lambda mutant is mildly sensitive to DNA double strand breaks but defective in integration of a transgene
Source: Front Plant Sci. 2015 May 27;6:357. doi: 10.3389/fpls.2015.00357 (PMC4444747; doi:10.3389/fpls.2015.00357)
Supplement: Supplementary file 4 [file Table2.DOC]

**Supplementary Table 2** Sensitivity of wild-type and mutants to BLM at 0.25 μg mL^-1^

|  |  | The ratio of swelling root tips (Average ± SD) | | |
| --- | --- | --- | --- | --- |
|  | Genotype | Days after transfer | | |
|  |  | 1 | 2 | 3 |
|  | WT Col | 0.00 ± 0.00 (53) | 3.11 ± 3.79 (53) | 83.34 ± 12.43 (53) |
|  | *atpol**λ-1* | 0.00 ± 0.00 (54) | 10.47 ± 3.63 (54) | 91.61 ± 13.66 (54) |
|  | *atlig4-2* | 0.00 ± 0.00 (44) | 100.00 ± 0.00 (44) | 100.00 ± 0.00 (44) |
|  | *atpolλ-1/atlig4-2* | 0.00 ± 0.00 (47) | 100.00 ± 0.00 (47) | 100.00 ± 0.00 (47) |
|  | *atku70-3* | 0.00 ± 0.00 (53) | 100.00 ± 0.00 (53) | 100.00 ± 0.00 (53) |
|  | *atku70-3/atpolλ-1* | 0.00 ± 0.00 (45) | 100.00 ± 0.00 (45) | 100.00 ± 0.00 (45) |

Seeds were imbedded in cold water for two days before irradiation and germinated on MS plates. The three day-old seedlings were transferred to MS plates supplemented with 0.25 μg mL^-1^ bleomycin. At each time point after transfer, the number of swelling root tips was scored by microscopic observation. Values are mean ± standard deviation (SD). Numbers in parenthesis indicate the total number of plants scored across all three replicates.
